# Asymptomatic amebiasis diagnosed 10 years after exposure to the pathogen

**DOI:** 10.1002/jgf2.527

**Published:** 2022-02-13

**Authors:** Takayuki Yamada, Kunihiro Hamada

**Affiliations:** ^1^ Internal Medicine Asunaro Clinic Takasaki City Japan; ^2^ Surgery Flash Hospital Fujioka City Japan

**Keywords:** amebiasis, colonoscopy, late diagnosis, trophozoites

## Abstract

A 43‐year‐old man underwent total colonoscopy for colorectal cancer screening. Colonoscopy revealed multiple erosions on the cecum. Biopsy specimens revealed multiple entamoeba trophozoite unexpectedly.

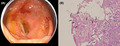

A 43‐year‐old man twice tested positive on an immunochemical fecal blood test (IFBT). Since the age of 40, he had undergone annual health checkups, which included having an IFBT. No abnormalities were noted in his past three medical examinations. He reported no gastrointestinal symptoms or weight loss, and he was taking amlodipine for essential hypertension. During colonoscopy screening for colorectal cancer, multiple cecal erosions with white moss‐like lesions of approximately 10 mm in diameter (Figure 1A) were observed. The differential diagnosis included early colon cancer or ulcerative colitis, and a diagnostic biopsy was performed. The biopsy results indicated multiple *Entamoeba* trophozoites attached to the colonic epithelium, with regenerative glands and a chronic diffuse inflammatory reaction in the lamina propria (Figure 1B). Chest and abdominal computed tomography scans did not show any lung or liver abscesses. Serum markers for human immunodeficiency virus (HIV), hepatitis C virus, and hepatitis B virus were negative.

**FIGURE 1:**
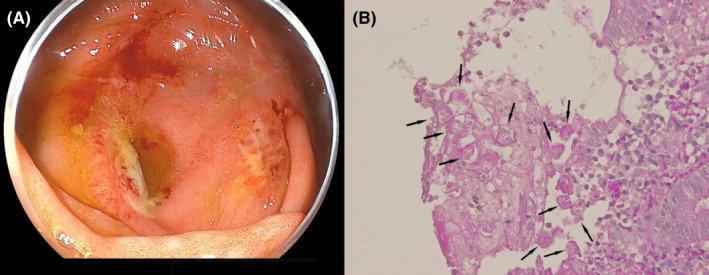
(A) Colonoscopy showing multiple cecal erosions of approximately 10 mm in diameter with a white moss‐like appearance. (B) Biopsy specimen showing multiple *Entamoeba* trophozoites attached to the colonic epithelium, with regenerative glands and a chronic diffuse inflammatory reaction in the lamina propria (PAS stain)

On further questioning, the patient reported no history of sexual relations with male partners or with commercial sex workers. However, 13 years ago, he visited Thailand and Cambodia over a 2‐year period. While staying in these countries, he had taken care with his diet and had avoided drinking local water. However, in Thailand, he experienced flooding twice during typhoons and reported that he accidentally drank muddy water and had developed diarrhea.

The patient was diagnosed with intestinal amebiasis >10 years after exposure during his long‐term business trips. He was treated with paromomycin for 10 days, with no adverse reaction. He was scheduled to have a follow‐up colonoscopy in 6 months to confirm disappearance of the lesions.

The cecum is a favored site for *Entamoeba* infection.^1^ The prevalence of amebiasis is low among the general population in industrialized countries, including Japan,^2^ and a pathological diagnosis is unexpected. In industrialized countries, *Entamoeba* infection is largely confined to subpopulations with anal sexual contact or HIV infection.^3^ This patient was HIV‐negative and did not report having partaken in any high‐risk sexual behavior.

Amebiasis resembles inflammatory bowel disease on colonoscopy.^4^ If trophozoites had not been detected in the biopsy specimen, the patient would have been diagnosed with ulcerative colitis. Thus, in situations where inflammatory bowel disease is considered in colonoscopy, it is important to take into account colonic amebiasis and obtain a detailed exposure history.

## CONFLICT OF INTEREST

The authors have stated explicitly that there are no conflicts of interest in connection with this article.

## INFORMED CONSENT

Informed consent was obtained from the patient for the publication of the case details.
